# Investigation of the performance of a cylindrical hopper and metering device of a carrot seeder

**DOI:** 10.1038/s41598-022-25798-8

**Published:** 2023-01-16

**Authors:** Marvin T. Valentin, Andrzej Białowiec, Davut Karayel, Algirdas Jasinskas, Daniel Ciolkosz, Jeffrey A. Lavarias

**Affiliations:** 1 Wrocław University of Environmental and Life Sciences, Department of Applied Bioeconomy, 25th Norwida Str. 51-630, Wrocław, Poland; 2 Associate Member, Engineering and Industrial Research, National Research Council of the Philippines, Department of Science and Technology, Taguig, Philippines; 3 Benguet State University, Km. 5, La Trinidad, 2601 Benguet, Philippines; 4 Department of Agricultural Engineering and Safety, Vytautas Magnus University, Agriculture Academy Studentu 15A, 53362 Akademija, Kaunas Reg., Lithuania; 5 Faculty of Agriculture, Department of Agricultural Machinery and Technologies Engineering, Akdeniz University, 07070 Antalya, Turkey; 6 Department of Agricultural and Biological Engineering, Pennsylvania State University, University Park Campus, State College, USA; 7 Department of Agricultural and Biosystems Engineering, College of Engineering, Central Luzon State University, Science City of Muñoz, Nueva Ecija, Philippines

**Keywords:** Mechanical engineering, Applied mathematics, Engineering, Mathematics and computing

## Abstract

A carrot seeder that utilizes a cylindrical component serving as both hopper/metering device to deposit seeds precisely into planting beds/hills at a uniform interval along a straight row over a plant bed was developed in this study. The seeder was evaluated at different operating speeds (89, 70, 61, 51, 48, 38, and 34 cm s^−1^) relative to mean number of seeds planted in each hill, hill center, scattering distance ratio, hill spacing and missed hills. The seeder was able to deposit seeds in each hill ranging from 2.8 to 4.0 at all speeds. The mean hill center and missed hills suggested that the seeder is best operated at speed under 70 cm s^−1^ with the respective values ranging from 0.9 to 1.6 cm and 0–5.5%. This indicates that number of the seeds dropped per hill was very uniform, which is a good indicator of the seeding performance. The hill center and missed hills at 89 cm s^−1^ were 3.08 cm and 16.67% respectively were significantly higher than at lower speeds. Other operating performances such as the mean number of seeds deposited in each hill and the distance between hills did not appear to vary with the planting speed. The scattering distance ratios of operating speeds from 34 to 61 cm s^−1^ were less than 30% and therefore acceptable for hill dropping of carrot seeds.

## Introduction

Carrot is a high-value crop commonly grown in the Philippine Highlands^1^ and Poland^2^ It grows well in the Cordillera Administrative Region (CAR), particularly in relatively cold places like the provinces of Benguet, Mountain Province, and Ifugao of which the province of Benguet being the largest producer^3^. Other provinces in the Philippines like Nueva Vizcaya, Cebu, Davao del Sur, Negros Oriental, and Bukidnon also produce carrots but in smaller quantities. In 2019, carrot production in the country was 65,069.67 MT, wherein 58,116.08 MT (89.31%) was harvested from CAR^4^. Additionally, the total farm area in 2019 cultivated for carrot production was 4550.09 ha, and 70.5% (3210.81 ha) of it was from CAR^4^. Poland is the third largest producer of carrot in Europe, accounting for 733,000 tons in 2020 (a 12% share)^2^.

In carrot farming, optimum crop yield is associated with proper seed germination along with other factors^5^. Optimum germination is noted to begin with planting the appropriate number of seeds into the desired spacing^5–7^. Carrots, when planted too close, have a lesser chance to develop optimum-sized roots^8^. Likewise, during planting, seeds should be planted at a suitable depth to avoid drying^8^. Additionally, too many carrot seeds planted in a hill would require extensive thinning^9^. This thinning operation is difficult to perform manually and entails added labor and cost^6^. This can also cause root damage to the remaining plant in a hill due to uprooting of nearby plants. Ideally, one carrot seed should be planted on each hill. However, in an open field, especially during rainy seasons, a single carrot plant emerging is susceptible to damage due to rain impact and insect attack. With this in mind, to avoid replanting, which is costly and leads to non-uniformity of plant growth, from actual observation, 2–6 seeds per hill are typically planted. FAO recommends a seeding rate of 6 kg ha^−1^ subject to prevailing environmental conditions^10^. Yehia et al.^11^, investigated the performance of a carrot seeder at different operating speeds at three cell sizes that included 3 seeds per cell using coated and uncoated seeds.

Carrot seeds are seeded directly to the plant bed^12^. This is because, once the carrot seed starts to germinate, its main root must not be disturbed or growth will be negatively impacted. The carrot seeds, when planted at optimum spacing where it will not be disturbed or be subjected to competition from other plants, will have the greatest chance to develop a marketable, high value root^13^. Hence, one of the important requirements for a carrot seeder is its ability to accurately deposit the seeds into the hills at a uniform spacing^8,14^.

A carrot-planting device should plant seeds in the shortest possible time in the field while still achieving uniform germination. One approach to accomplishing this is by increasing the capacity of the seeder’s effective working width^15^. The effective working width can be expanded to plant additional rows by adding metering discs to make it a multi-row seeder. However, this must be carefully considered because planting accuracy can be sacrificed at extremely high or low capacity^16^.

Seed damage and seed distribution efficiency are influenced by the rotational speed of the metering disc. Higher speed can reduce distribution efficiency and increase damage to seeds. This trend is shown in the findings of Ekka et al.^17^, where seed distribution efficiency of a jute seeder decreased from 91 to 86% as metering disc speed increased from 30 to 60 RPM. Relatively low seed damage of 3.51% was observed by Bamgboye and Mofolasayo^18^ at lower speed in a two-row okra planter in a laboratory test.

Another important parameter of a carrot seeder (yet difficult to measure) is the desired spacing between hills^19^. A seeder must deposit seeds close to the designed interval or spacing to maximize crop yield^20^. Yazgi and Degirmencioglu^21^, using response surface methodology (RSM), optimized the spacing uniformity of a precision seeder with a vertical metering plate by considering the different levels of peripheral speeds of the seed plate, vacuum pressures, and hole diameters. Peripheral speeds used in the operation of the seeder were 5.0, 8, 12.0, 16.0, and 19.0 cm s^−1^. The researchers concluded that the seeder had better performance at lower peripheral speeds as indicated by the percentage of missed hills, and quality of feed index^21^.

Singh et al.^19^, investigated the influence of different linear speeds of seed disc (29.0, 42.0, 58.0, and 69.0 cm s^−1^), vacuum pressure (1.0, 1.5, 2.0, and 2.5 kPa), and entry angles (90°, 120°, and 150°) of the seed hole using an optimized size of 0.02 cm seed cell diameter to the performance of a pneumatic cottonseed planting device relative to the average seed spacing and the precision of spacing. Additional parameters considered in the evaluation were skips or missed hills (miss index), multiple index, and the highest quality of feed index. They concluded that the optimum cone angle was 120^0^ that positively affected the performance of the seeder resulting in the lowest miss index and multiple index, and the highest quality of feed index. Average values of the plant spacing and precision spacing were influenced by the speed and pressure. The quality of feed index of the seed was highest (94.67%) at linear speeds at 42.0 cm s^−1^ but was statistically indistinguishable from a linear speed of 29.0 cm s^−1^. Further, the seeder had the lowest miss index of 1.33% and multiple index of 4.0% when the metering disc was operated at 42.0 cm s^−1^. The miss index had increased at increased speed as seed cells did not have sufficient time to load seeds.

Zhan et al.^14^, investigated seed spacing uniformity of a vacuum-cylinder precision seeder on rape seeds as affected by suction pressure and sowing angle with the use of numerical analysis in a laboratory setting. They also assessed the seeds’ free flight motion and the corresponding forces acting on seeds were analyzed with the aid of computational fluid dynamics (CFD). The falling trajectories of the seeds were recorded with the aid of a high-speed camera and a tracking device. They found out that, upon analyzing the seed falling trajectories, the seeding uniformity was affected by the release angle with optimum level of − 10 to 10°. The error associated with spacing was at a minimum at a release angle of 5°.

Ryu and Kim^7^ designed a precision planter with a roller-type metering mechanism and evaluated it at speeds ranging from 022.0 to 83.0 cm s^−1^ against the scattering distance ratio (SDR). It was found out in their study that the SRD at all speeds was 25 to 30% which was sufficient for the design to be considered as a precision planter.

With the aid of a speed detector camera system (Kodak Ektapro HS high-speed) under laboratory condition, Karayel et al.^22^, investigated the performance of a common seed drill unit on wheat and soybean seed in terms of fall velocity of seeds and seed spacing as influenced by the metering rollers’ rotation at 10, 20, 30, and 40 rpm over a constant operating speed of 100.0 cm s^−1^.A comparative evaluation was also made using a sticky belt. They observed that spacing, expressed in terms of the coefficient of variation, was more uniform at an increased speed of the metering rollers. The use of 40 rpm for both seeds in a seed drill was more accurate than 10 RPM.

Meanwhile, carrot producing regions in the Philippines, especially in the province of Benguet, are mountainous with small and fragmented farm holdings that pose challenges to the use of mechanization for tasks such as carrot seeding among others. Rasouli et al.^23^, noted that small farms and scattered land holdings are a major inhibition to mechanization.

This study aimed to design, for the first time, a multi row carrot seeding machine applicable to the highland regions of the Philippines. The working performance of the seeder was evaluated in terms of the number of seeds deposited in each hill, hill center, spacing between hills, Scattering Distance Ratio (SDR), and missed hills. The performance was analyzed as influenced by the forward operating speed in the laboratory and in field conditions following methodologies adopted elsewhere^19,22,24^.

## Materials and methods

### Seed

A group of 100 uncoated carrot seeds (Tokita Kuroda carrot seeds), 2.2 g 1000^–1^ seeds^−1^, were randomly selected from carrot seeds purchased from a local agricultural supplier. These samples were subjected to laboratory measurement and the resulting values were recorded (Table 1). The measurement was done with the aid of a caliper and digital weighing balance with respective sensitivity values of 0.005 mm and 0.01 g.

**Table 1:** The physical properties of carrot seeds (uncoated) used in the laboratory of the carrot seeder.

Physical properties	Unit	Mean	SD	CV, %
Length $l$	mm	3.85	0.04	1.10
Width $w$	mm	1.72	0.02	1.09
Thickness $t$	mm	0.80	0.03	4.34
Sphericity, ^a^	%	45.32	0.41	0.90
Geometric mean diameter^b^	mm	4.63	4.63	2.09
Thousand seed mass	g	2.20	–	–

^a^Calculated as $$\frac{{\left( {lwt} \right)^{1/3} }}{l} \times 100\%$$^14,21^.

^b^Calculated as $$\left( {lwt} \right)^{1/3}$$^19,25^.

### Design considerations

The objectives for the design of the carrot seeder considered several aspects such as the number of seeds to be planted in each hill, portability due to farm size and topography, number of rows, and distance between rows and hills as currently practiced by farmers. The designed number of seeds to be deposited in a hill should not exceed 6 seeds as calculated based on the recommended plant density^10^. The spacing between rows and hills was set at 5.0 cm and 17.0 cm, respectively. Furthermore, considering the farm size and topographic conditions wherein the farmer needs to hand carry the seeder to transfer to the next farm lot or terrace, the overall weight of the seeder has to match the carrying capacity of a farmer. Therefore, the design weight must not exceed 12.0 kg.

### Metering design

**Figure 1:**
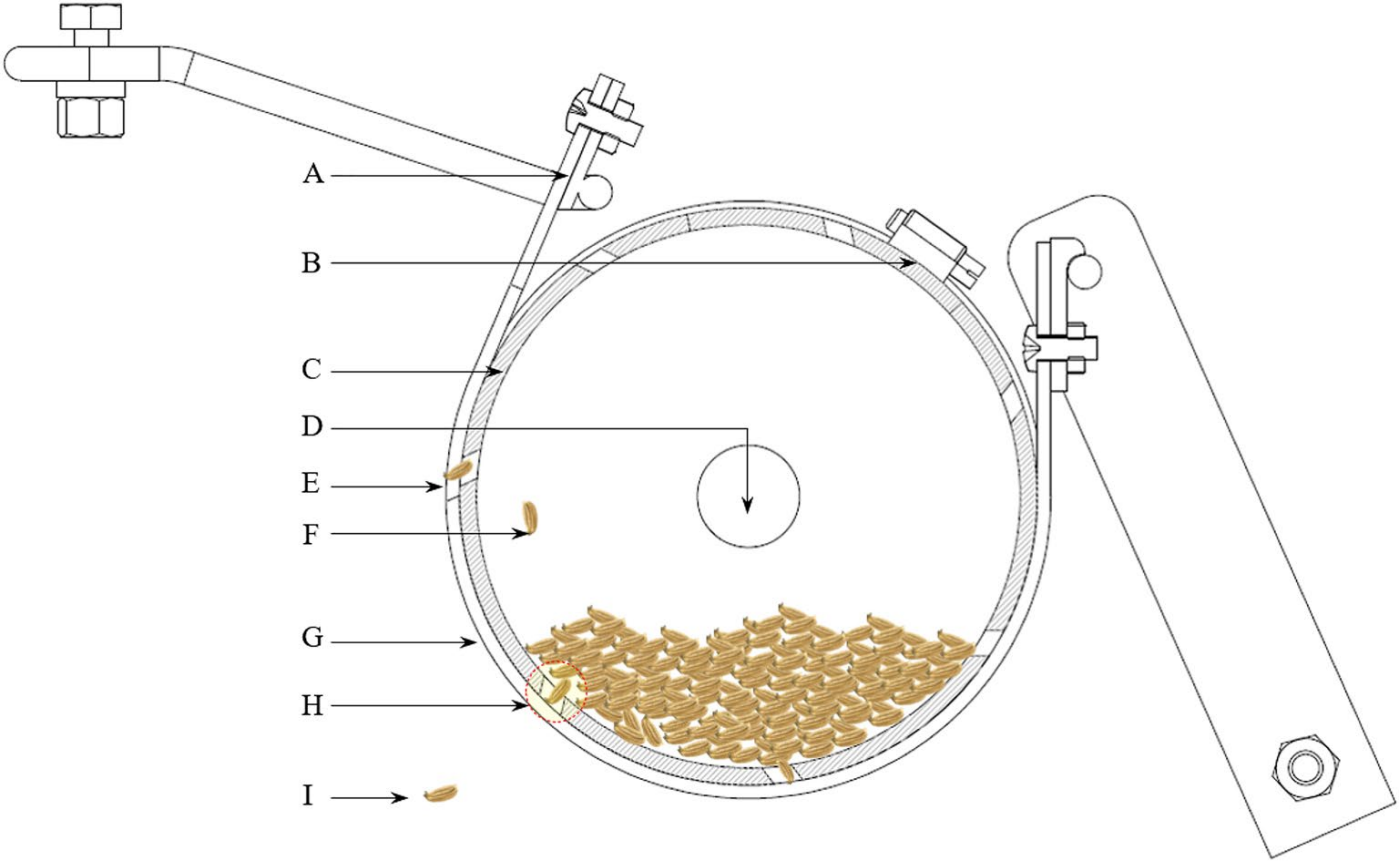
Schematic diagram of the metering assembly with features as (**A**) rigid frame that holds the strap and the cylinder through bolts; (**B**) lock mechanism of the cylinder; (**C**) metering cylinder; (**D**) shafting through which the metering cylinder rotates; (**E**) hole discharge point of the strap showing a seed about to fall into the ground; (**F**) excess seeds from the seed cell falls back into the cylinder; (**G**) strap; (**H**) seed cell inclined at 45°; and (**I**) discharged seed.

The metering component of the seeder is a cylinder that serves as both hopper and metering device (Figs. 1 and S3). The cylinder has a series of holes around the circumference serving as seed cells that collect seeds and dispense them into the ground through the designated discharge point. There are 7 seed cells around the circumference of the metering cylinder. The seed cell is designed to accommodate 2 to 6 seeds as detailed below. A strap is provided around the circumference of the metering cylinder covering a quarter of its circumference which works as seed stopper so that seeds will fall to the ground at the intended time. The seed discharge point is located on the upper portion of the cylinder so that excess seeds in the seed cell fall back into the bottom portion of the cylinder before they reach the discharge point.

### Seed cell design

**Figure 2:**
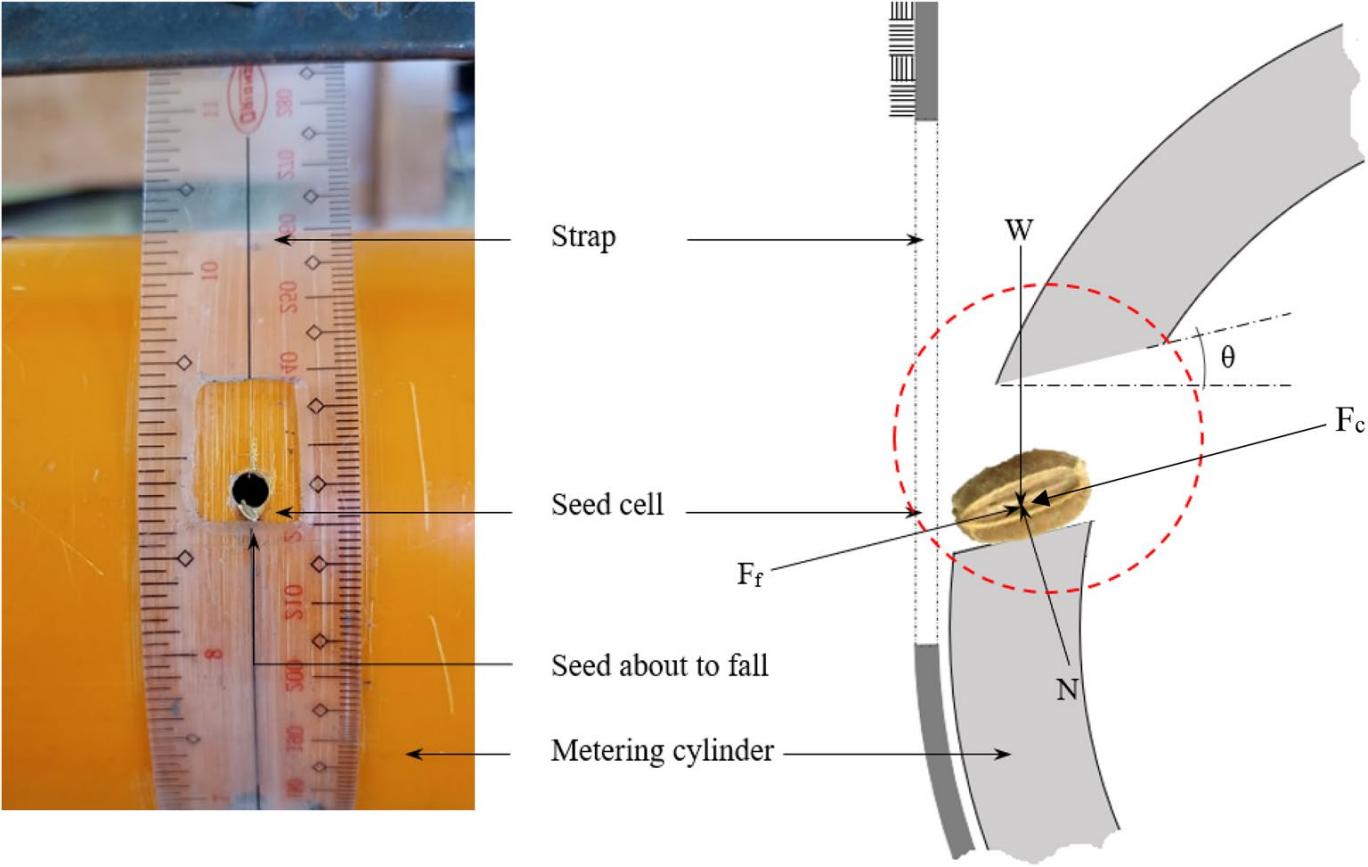
Schematic diagram of the fabricated metering cylinder showing the seed cell focusing on the seed that is about to fall out of the seed cell at the discharge point and the accompanying forces acting on the seed; W, the weight of the carrot seed; N, the normal force acting perpendicular to the inclination, and F_f_, the frictional force of the seed to the metering cylinder.

The size of the seed cell in terms of the angle of inclination, the diameter, and the height was designed relative to the desired number of seeds it would contain which is 2 to 6 seeds. The volume of the seed cell was calculated on the basis of the projected volume of 6 carrot seeds, which is the maximum amount of the seed the cell should contain. Considering the possible orientation of a seed in the cell, the scenario by which the seed at the exit point would either fall out of the cylinder or not is illustrated by the equilibrium condition shown in Fig. 2. The cylinder used was a PVC pipe with 10.16 cm diameter, 25 cm effective length and thickness of 0.05 cm. The strap used was a flexible transparent plastic.

At the designated point for the seed to fall out of the cylinder, considering the possible forces affecting the fall of the seed gives a working equation to estimate the angle of inclination of the seed cell (Eq. 1).

1 $$\theta = \sin^{ - 1} \left( {\frac{{F_{f} - F_{c} }}{\mu mg}} \right)$$

where ${F}_{c}$ is the centrifugal force due to cylinder rotation, ${F}_{f}$ is the frictional force that impedes the seed to slide, $\theta$ is the angle of inclination of the seed cell, $\mu$ is the static friction coefficient (SFC) of seed on the cylinder, $m$ is the mass of the seed, and $g$ is the acceleration due to gravity. The most favorable condition for the seed to freely slide from the cylinder is when the frictional force is minimal. This force can be modified by increasing the angle of inclination of the seed cell. A plane with adjustable inclination was used to determine the SFC adapting previously published works^26,27^. A set of 50 seeds were used in the experiment. Each seed was placed on a plane made of polyvinyl chloride material and the plane was gradually inclined from its horizontal position until the seed started to slide. The angle in which the seed started to slide was recorded as the angle of inclination. The coefficient of friction and the angle were calculated using Eq. 2 as adopted by Kaliniewicz et al.^28^

2 $$u = \tan \theta$$

**Figure 3:**
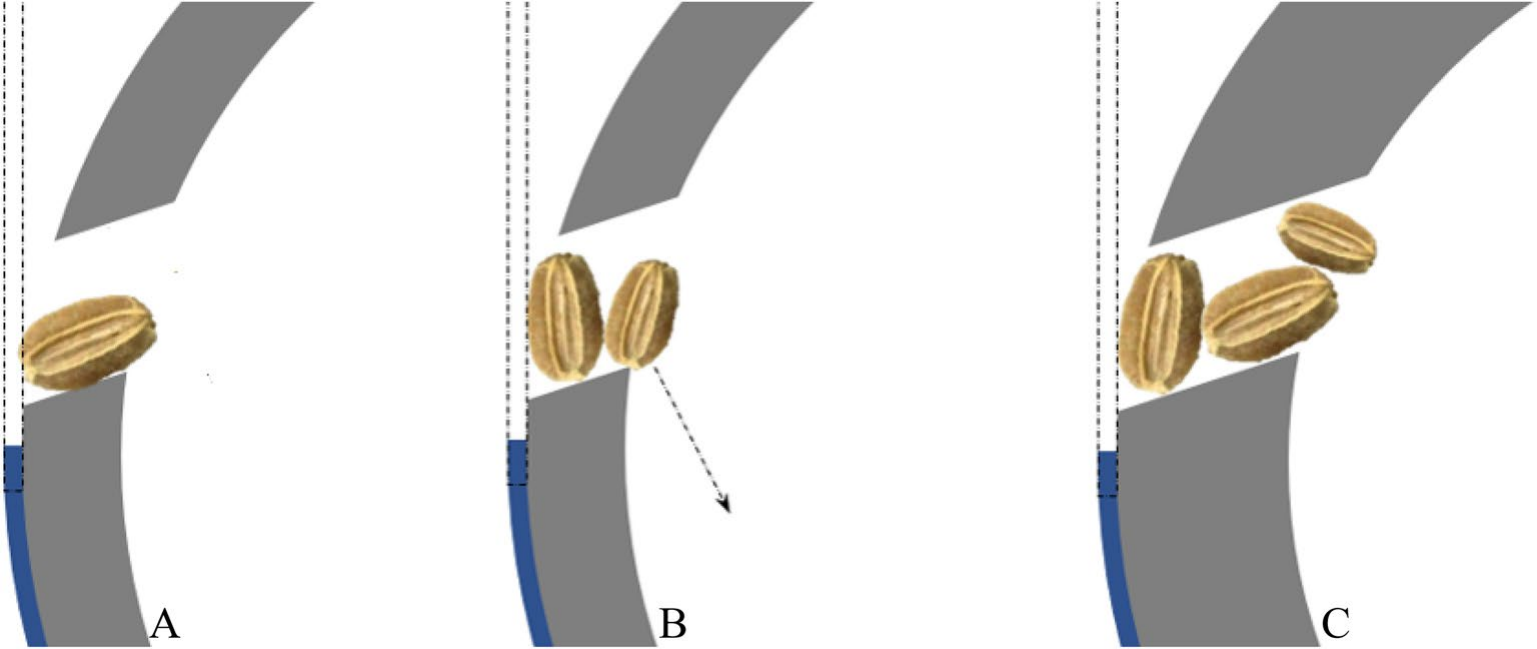
Possible seed loading scenarios of carrot seeds into the seed cell, (**A**) the seed is loaded to the cell at slant position; (**B**) seed is loaded at the vertical position; and (**C**) seed cell with larger thickness.

The angle of inclination, $\theta$, is a critical design parameter of the seed cell. This affects the regular dropping of seeds from the cell itself^7^. The diameter of the seed cell, $d$, must be slightly greater than the seed length for the seed can be loaded at either slant or vertical position. The slant height, $sh$, of the seed cell is a function of the thickness of the metering cylinder and the angle of inclination, $\theta$. At 90 degrees, $sh$ is the same as the thickness of the cylinder but at angles lower or higher than 90 degrees, the slant height begins to increase. At smaller $sh$, the cell can still load seed even if a portion of it is hanging (Fig. 3A). The seed in this scenario can still be carried by the metering cylinder to the discharge point during rotation provided that most of its length is in the seed cell; otherwise, the seed will fall back in the hopper. If the thickness of the cylinder is too small, the seed cell may not be able to load seed especially those at slant position but it can still load seeds that are in a vertical position. However, it is not always that seeds are oriented in an upright position when loaded to the cell. Therefore, some will be loaded while at a slant orientation. This loading scenario when the slant height of the seed cell is too small may contribute to missed hills.

The size of the seed cell is a critical consideration especially that the carrot seeds are not uniform in size. For the optimum condition, the seed cell design considered the largest size of a carrot seed from the sample. An allowance in the size of the seed cell with reference to the seed size was considered to avoid possible clogging especially when the seed is closely fitted to the cell which will affect the discharge efficiency. This means that the seed cell should be able to load the minimum desired number of large-sized seeds. Consequently, when the seeds are below the average size, then the seed cells will have the potential to load a higher number of seeds at one time. From these premises and considering the recommended plant density of 6 kg ha^−1^^10^, 5–10 kg ha^−1^^1^ germination rate of the seeds and factoring hill and row spacing (Fig. S1), calculation indicates that 6.0 carrot seeds per hill is sufficient as presented in the sec 2 of supplementary material. Similar suggestion was also obtained from local farmers that at least 6 seeds can be planted per hill. The size of the seed cell was then selected to accommodate a maximum of 6 seeds which was also validated and adjusted at the laboratory.

The diameter of the seed cell should also be slightly bigger than the seed length. If the diameter is too big then the chance of the seed cell to load a greater number of seeds is high, and would contribute to variability in the mean number of seeds dropped in each hill. If the seed is loaded at a vertical position in the cell, there could be space for another seed to be accommodated (Fig. 3B). However, if this space is too small such that it cannot support another seed, then the excess seed will fall back to the cylinder. If the seed will always be loaded in the cell at a slant position, then the diameter should be slightly larger than the width of the seed, but this is not possible since the seed loading position is not regulated. Otherwise, this will contribute to missed hills. The thickness of the cylinder affects the slant height of the seed cell and should be a bit smaller than the average seed length. At larger thickness, the cell will have the chance to load more seeds even if the seeds are at a slant position (Fig. 3C).

**Figure 4:**
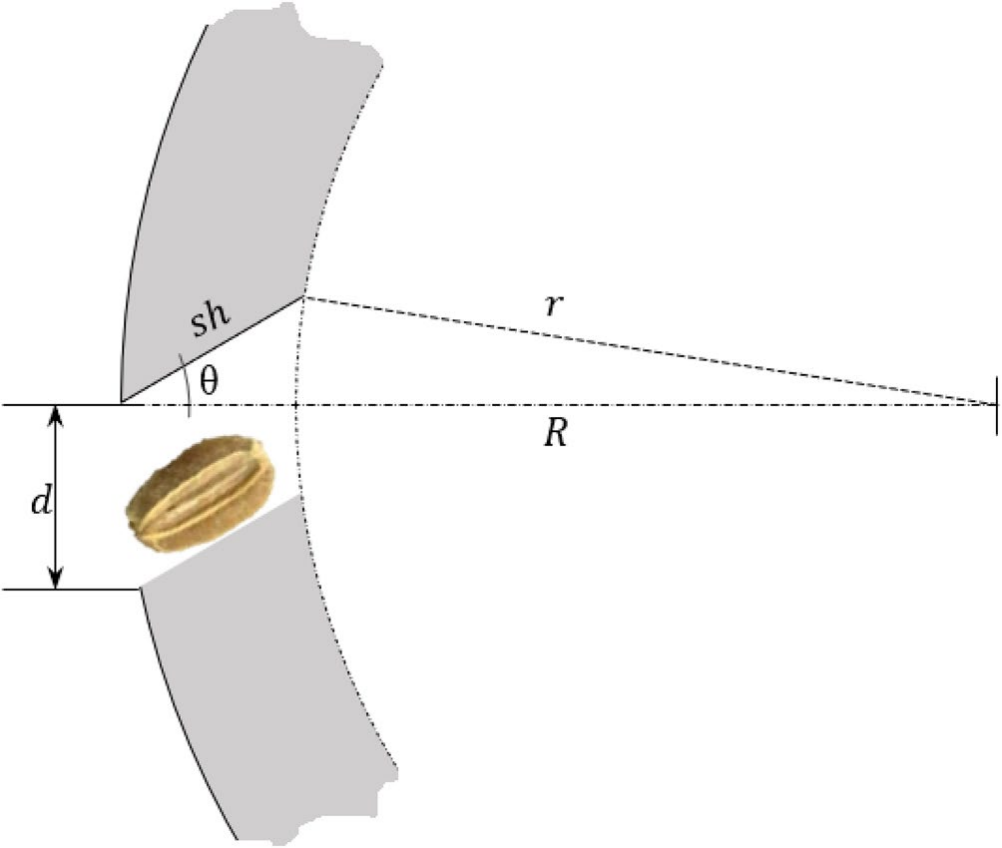
The variables comprising the seed cell size, diameter of the seed cell, *d*; depth or slant height of the seed cell, *sh*; angle of inclination which is the angle to which the seed starts to slide off the seed cell surface, θ; inside radius, r; and outside radius, *R*.

The size of a seed cell as depicted in Fig. 4 consists of the cell diameter, d, the slant height or the depth of the cell, $sh$, which is inclined at $\theta$ or this is the angle by which the seed starts to slide off the surface of the cell. Higher values of the angle of inclination are better for the seeds to slide or escape quickly out of the seed cell. The thickness of the cylinder is the difference between the outside radius, $R$, and the inside radius, $r$.

Applying the law of cosine, the relationship of the dimensions of the seed cell described in Fig. 4 is given in Eq. (3):

3 $$r^{2} = sh^{2} + R^{2} - 2R\left( {sh} \right)\cos \theta$$

The dimension of the seed cell affects its seed loading efficiency. The value of the slant height, $sh$, should not exceed the mean seed length $l$ which was experimentally determined as 3.8 mm (Table 1) but it should would be large enough to accommodate most of the seed to avoid its falling back into the seed chamber. The slant height was designed for this condition following an approach developed elsewhere^29^. In this study the limit of the slant height was set at 50–80% of the seed length.

4 $$0.5l < sh < 0.8l$$

Similarly, the diameter,$d$, of the seed cell was calculated using:

5 $$d = 0.8 \left( l \right)$$

The thickness, $t_{c}$ of the cylindrical metering drum was determined by substituting Eqs. (1), (4), and Eqs. (5) to (3):

6 $$t_{c} = R - r$$

The number of seed cells in the metering cylinder is a function of the spacing between hills of seeds on the ground and was determined using Eq. (7).

7 $$n = \frac{{\pi D_{c} }}{iHS}$$

where $n$ is the number of seed cells around the metering cylinder, $D_{c}$ is the diameter of the metering cylinder in $cm$, $i$ is the transmission rate between wheel and metering cylinder and $HS$ is the recommended spacing between hills for carrots which is 17.7 cm.

The diameter of the metering cylinder was also determined in relation to its optimum equivalent linear velocity. A smaller diameter would allow the metering cylinder to rotate at a faster rate which could damage the seeds and could induce an “avalanche”^30^ movement of the seeds such that seed layer height above the seed cell becomes irregular and the seeds do not have enough time to be loaded into the seed cells. Also, the size of the cylinder is related to its capacity to hold a quantity of seeds that relates to frequency of refilling during the planting operation. Frequent refilling as a result of a smaller metering cylinder can affect the planting capacity of the seeder. Cylinder maximum filling capacity was set to 80% of its volume in this study. On a basis of 0.5 ha with hill spacing of 17.7 cm and row spacing of 5.0 cm and considering the possible maximum number of seeds the seed cell can accommodate to be planted per hill which is 6 seeds, the metering cylinder must carry approximately 34,000 seeds so that refill would only be done every 0.5 ha of area planted. The volume of the seeds in the cylinder and the volume of the metering cylinder was then related by Eqs. (8) and (9). The mass of seeds used in this calculation included a small number of residual seeds remaining (20.0 g) after planting 0.5 ha. This is to provide a practical margin for error and ensure good performance of the seeder through to the end its 0.5 ha capacity.

8 $$V_{s} = \frac{{m_{s} }}{\rho }$$

9 $$V_{s} = 0.80V_{c}$$

where $V_{s}$ is the maximum volume of carrot seeds in the metering cylinder, $m_{s}$ is the measured equivalent mass of the carrot seeds in the metering cylinder, and $\rho$ is the equivalent bulk density of carrot seeds.

Considering the actual volume of the metering cylinder in relation to the maximum capacity of seed it can contain, Eqs. (8) and (9) results in Eq. (10).

10 $$D_{c}^{2} = \frac{{0.2V_{s} }}{\pi }$$

Meanwhile the linear velocity of the cylinder is given by Eq. (11)

11 $$v_{c} = \frac{{\pi D_{c} }}{t}$$

where $v_{c}$ is the linear velocity of the metering cylinder measured at its outermost circumference, $D_{c}$ is the diameter of the cylinder, $t$ is the time associated to the rotation of the metering cylinder. Furthermore, the amount of force needed to rotate the cylinder that will give an estimate of the force that must be exerted through the handle of the seeder to rotate the cylinder as it overcomes the friction developed between the cylinder and the stopper is related by Eqs. (12) and (13).

12 $$F = m_{mc} a$$

13 $$Fr = N\mu ; = m_{mc} g\mu$$

where $F$ is the force required to rotate the metering cylinder, $m_{mc}$ mass of the metering component, $g$ is the acceleration of the metering cylinder, $Fr$ is the frictional force developed between the metering cylinder surface and the seed stopper, $N$ is the normal force acting perpendicular to the surface of the metering cylinder, and $\mu$ is the coefficient of friction developed between the metering cylinder and the seed stopper. Considering that the magnitude of force to rotate the cylinder is equivalent to the frictional force then a useful relationship relating the acceleration of the cylinder is introduced in Eq. (14).

14 $$a = g\mu$$

The acceleration of the metering cylinder can be further related to its initial and final velocity over time defined in Eq. (15)

15 $$v_{f} = v_{o} + at$$

where $v_{f}$ is the final velocity of the metering cylinder, which is simply its working velocity, $v$, $v_{o}$ is the initial velocity of the metering cylinder, which is zero at the start of the rotation, $t$ is the time of rotation of the metering cylinder which can be customarily considered to be one rotation. Relating Eqs. (14) to (15) gives additional detail to the diameter of the metering cylinder in Eq. (16).

16 $$D_{c} = \frac{{gut^{2} }}{\pi }$$

Combining Eqs. (13) and (16) gives

17 $$\frac{{0.2V_{s} }}{\pi } = \frac{{g^{2} u^{2} t^{4} }}{{\pi^{2} }}$$

Equation (17) as expressed in terms of the volume and diameter of the metering cylinder would result to Eq. (18).

18 $$0.16V_{c} = \frac{{g^{2} u^{2} t^{4} }}{\pi }$$

Finally, the diameter of the cylinder that relates to its linear velocity and coefficient of friction is given in Eq. (19). The length of the cylinder is defined by the standard width of a plant bed as basis for the width of the seeder.

19 $$D_{c} = \frac{{25g\mu t^{2} }}{\pi }\sqrt L .$$

### Seeder assembly

**Figure 5:**
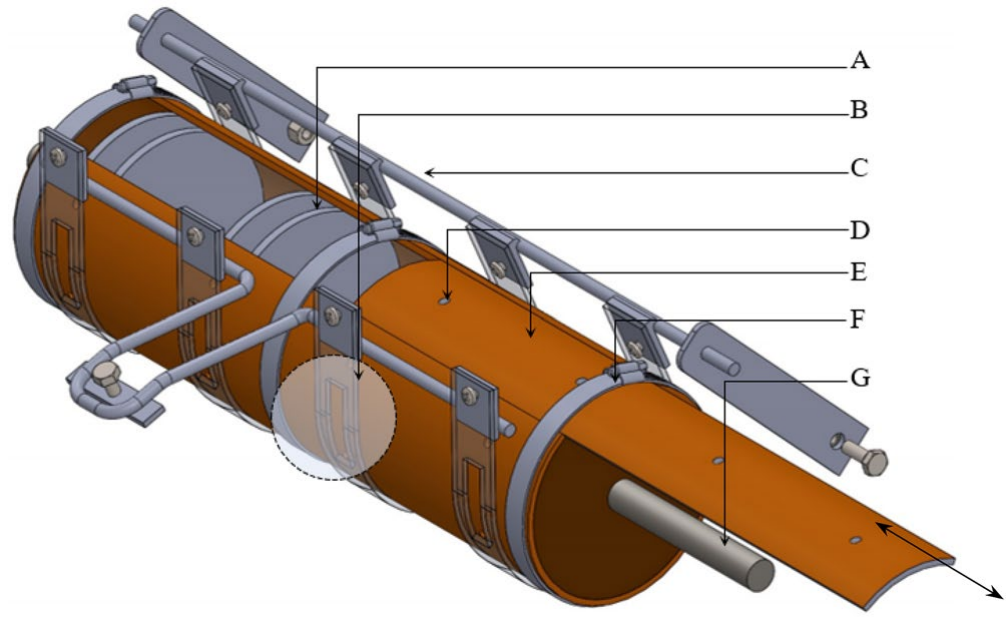
Schematic diagram of the metering assembly of the carrot seeder; (**A**) seed compartment; (**B**) seed discharge hole as earlier illustrated in Fig. 4; (**C**) frame assembly with bolts and nuts; (**D**) seed hole around the circumference of the cylinder; (**E**) removable cover for loading of seeds and removal of excess seeds; (**F**) locking component to lock the removable cover; and (**G**) axle to which the metering cylinder rotates.

The metering assembly comprised of a cylindrical drum, seed compartment, strap, axle, and frame is shown in Fig. 5. A compartment is provided inside the cylinder to hold the seeds for each individual seed row. The strap which serves as a seed stopper was made of transparent flexi glass with a thickness of 0.2 cm and a width of 2.50 cm. Four straps were prepared and were held together by the frame using bolts and nuts. A removable cover, locked using a girt screw, is provided to allow seed refill and removal of excess seeds after the operation.

**Figure 6:**
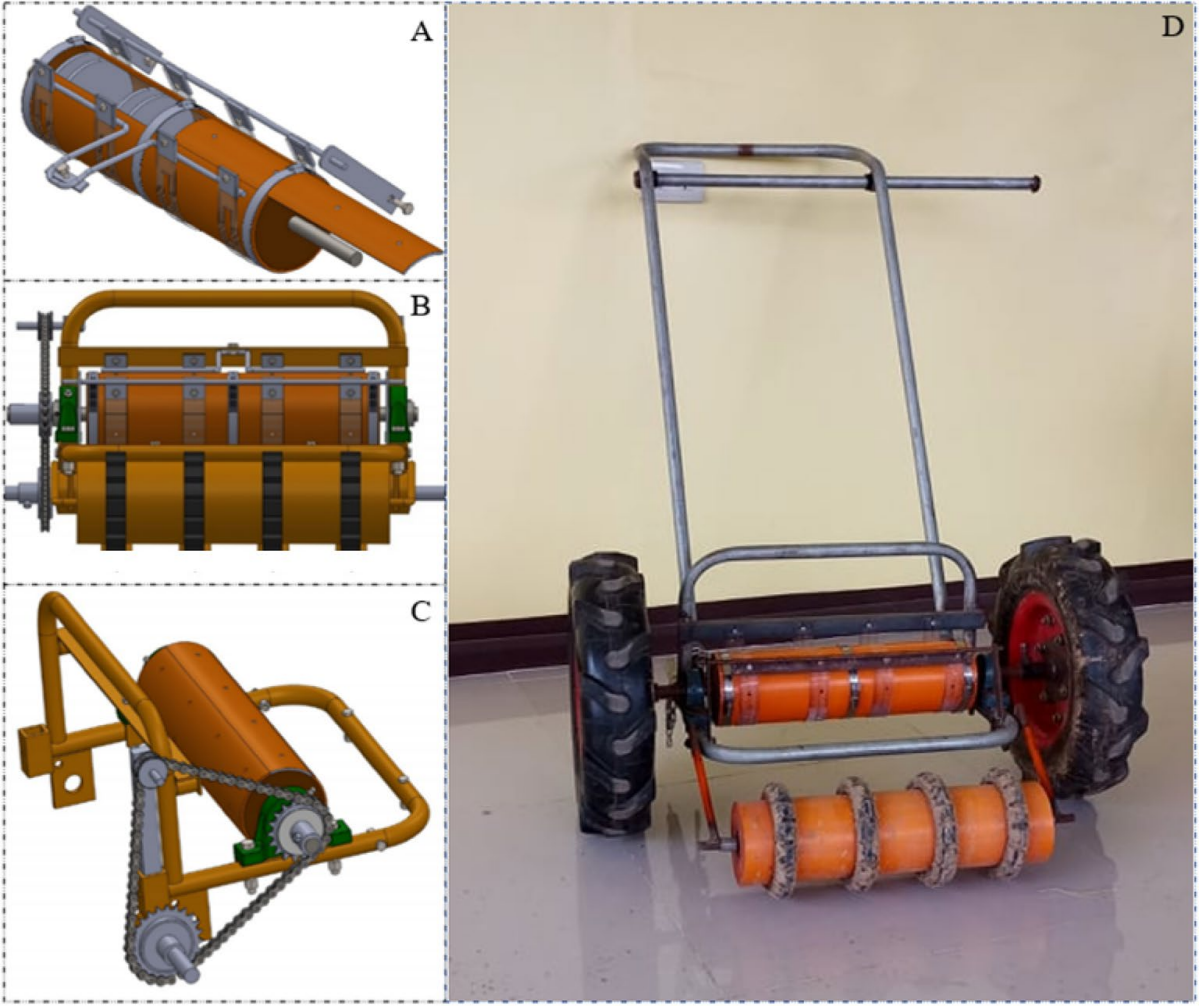
Carrot seeding device that was fabricated and tested in this study showing the (**A**) metering assembly as described in Fig. 3; (**B**) Furrow opener with a wheel to control the depth of furrow opening; (**C**) chain and sprocket assembly that transmits rotational motion from the wheel to the metering assembly; and (**D**) the actual picture of the fabricated carrot seeder.

The metering assembly was mounted in a complete seeding assembly (Fig. 6) and was subjected to evaluation in this study. There are four girths made from rubber that are rolled on the front cylinder serving as furrow openers into which the seeds are planted. These girths have a width of 2.54 cm and a thickness of 3.0 cm and are aligned with the seed cells. The cylinder to which the girths are installed helps maintain uniform depth of the furrow aside from serving as a balance component.

### Evaluation

The seeder was evaluated at different operating speeds, running each treatment in triplicate (Table 2). The speeds were selected by having seven operators operate the seeder at their individual “normal speed”. Different operating speeds were evaluated to test if the seeder will maintain its performance when operated by different operators at different operating speeds since not all farmers use the same speed. During the testing, the operator was allowed first to operate the seeder over the first 2.0 m of the plant bed to stabilize speed after which the timer started. The duration of each test was timed and was used to calculate the speed. A box that mimicked the real plant bed for carrot seed production was used with a dimension of 1000.0 cm long, 75.0 cm wide and 14.0 cm high. The surface of the box was coated with grease to avoid the seed bouncing when it falls from the metering cylinder as described elsewhere^6,12^. The field evaluation was conducted in an upland soil condition prepared by manual tilling to form a raised seedbed. The soil had a moisture content of 32.99% and bulk density 903.32 kg m^3^ as characterized by the Agricultural Machinery Testing and Evaluation Center (AMTEC)^31^.

**Table 2:** The experimental setup of the laboratory evaluation for the performance of the developed mechanical carrot seeder.

Sr. no	Treatments	Levels
1	Operating speeds (OS), cm s^−1^	OS_1_ = 89
		OS_2_ = 70
		OS_3_ = 61
		OS_4_ = 51
		OS_5_ = 48
		OS_6_ = 38
		OS_7_ = 34
2	Replication	3.0
3	Fall height, cm	14.0
4	Bed length, cm	1000.0
5	Bed width, cm	75.0

**Figure 7:**
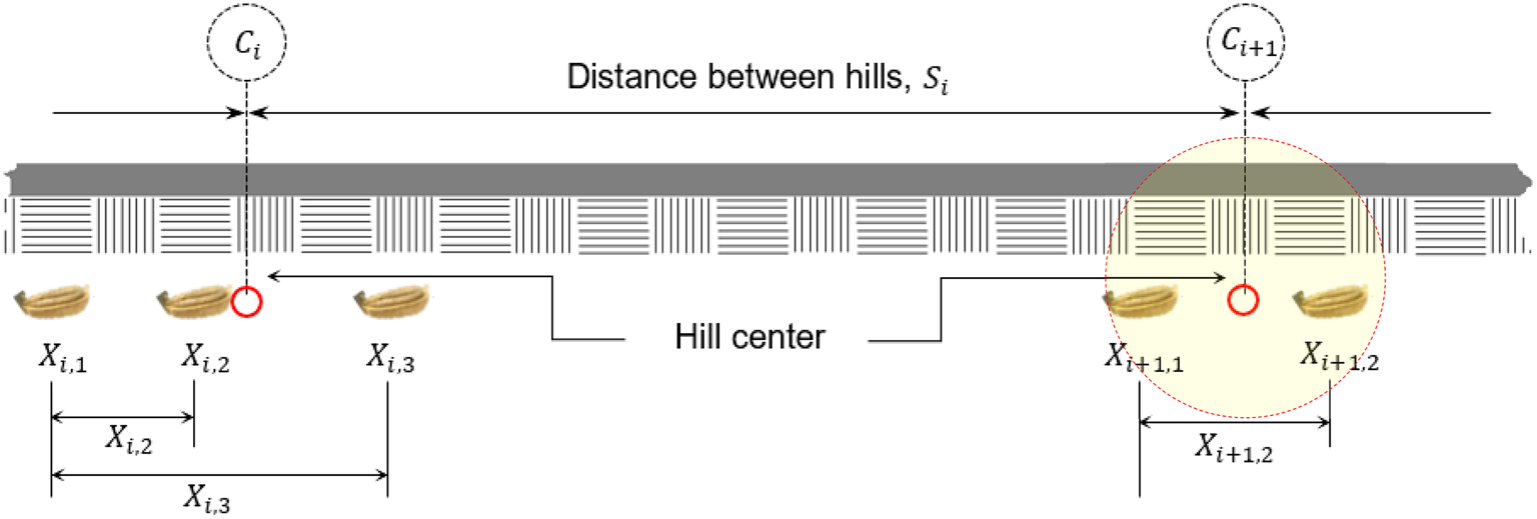
Schematic diagram of seeds sowed showing the position of each seed relative to the first seed dropped (*X*_*i*,1_) in a hill and the hill center as described in the largest circle inscribing the seeds in a hill.

The hill dropping uniformity was analyzed following the method described elsewhere^7,20,32,33^. The center position of seeds in a hill and distance between hills were measured and were used to determine the seed position, the dispersion ratio, and the scattering distance ratio (SDR) following the methods described by Ryu^7^ and Kim and Topakci et al.^33^, which were calculated using Eq. (7) through Eq. (10). The locations of the scattered seeds in a hill were measured relative to the seed that was first dropped in the hill. Seed location is assigned as $X_{i, j}$ in which subscripts $i$ and $j$ indicate the $jth$ seed in the $ith$ hill (Fig. 7).

The hill center of the seeds deposited in each hill was measured by taking the center of the circle inscribing all the seeds in a hill on a horizontal plane. This was simplified by measuring the distance between the first seed dropped into the hill. The hill center of the seeds in the ${\text{i}}th$ hill, $C_{i}$ was computed using Eq. (20).

20 $$C_{i} = \frac{{\mathop \sum \nolimits_{j = 1}^{{n_{i} }} X_{i, j} }}{{n_{i} }}$$

where $n_{i}$ is the sum of the number of seeds deposited in the $ith$ hill. The distance of the seed in the $ith$ hill is denoted as $X_{i,j}$ to the calculated hill center, $C_{i}$ and its ratio to the succeeding distance between hills $S_{i}$ is the position of the seed calculated using Eq. (21).

21 $$SP_{i,j} = \frac{{X_{i,j} - C_{i} }}{{S_{i} }}$$

The seeder performance in depositing seeds in a hill is also be described employing scattering distance ratio (SDR) and was calculated using Eq. (22) and (23).

22 $$SDR = 3.3\sigma \left( {SP} \right)100$$

23 $$\sigma \left( {SP} \right) = \sqrt {\frac{{\mathop \sum \nolimits_{i = j}^{m} \mathop \sum \nolimits_{j = 1}^{{n_{i} }} \left( {SP_{i,j} } \right)^{2} }}{{\mathop \sum \nolimits_{i = 1}^{m} n_{i} }}}$$

where $\sigma \left( {SP} \right)$ is the standard deviation of the measured distance between the seed and hill center, and $m$ is the total number of hills. The coefficient of variation of the spacing between the hills was calculated using Eqs. (24) and (25).

24 $$SD = \sqrt {\frac{{\mathop \sum \nolimits_{i = 1}^{N} \left( {x_{i} - \overline{x}} \right)^{2} }}{N - 1}}$$

25 $$CV = \frac{SD}{{\overline{x}}}$$

where $SD$ is the standard deviation, $\overline{x}$ is the theoretical seed spacing between two consecutive hills in the row which is 17.7 cm, $x_{i}$ is the measured distance between hills, $N$ is the total number of distances measured, and $CV$ is the coefficient of variation.

**Figure 8:**
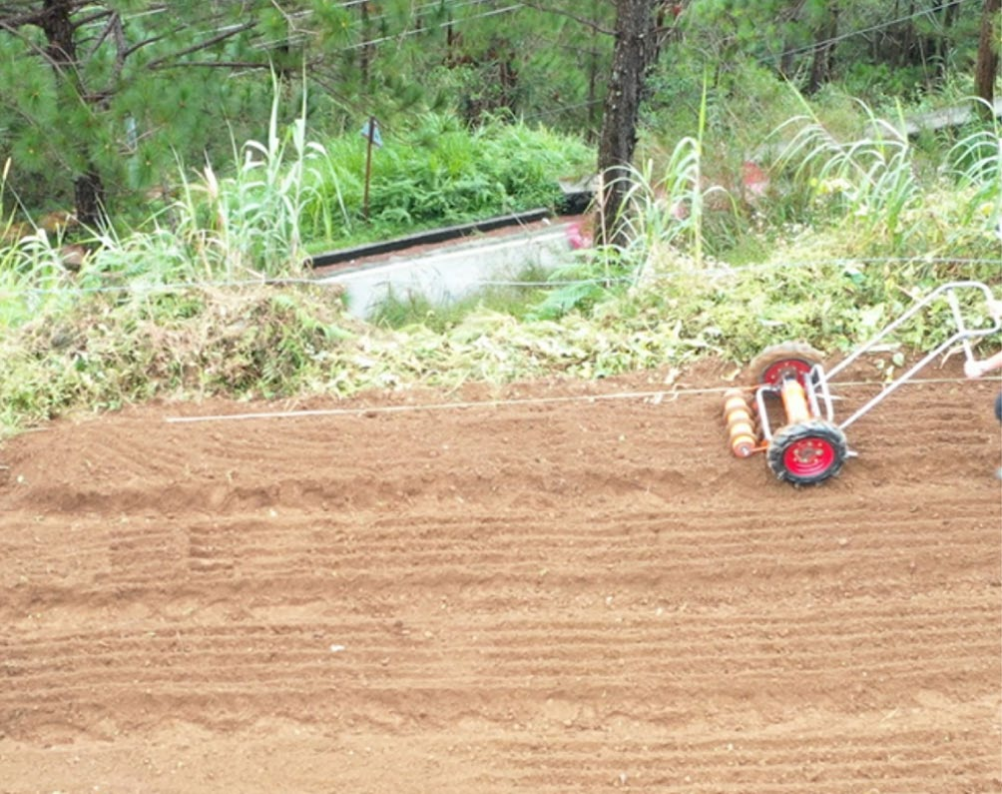
Field testing of the carrot seeder administered by the Agricultural Machinery and Testing Evaluation Center, University of the Philippines Los Baños (AMTEC-UPLB).

The guidelines provided by the Agricultural Machinery and Testing Center (AMTEC) and the Philippine Agricultural Engineering Standards for the specifications and method of testing seeders and planters were also observed during the field evaluation of the seeder (Fig. 8).

### Statistical tool

The recorded data on the number of seeds in each hill, hill center, spacing between hills, percentage missed hills, and scattering distance ratio were analyzed using analysis of variance (ANOVA). LSD was used to determine significantly different means within the performance parameters.

## Results and discussion

The performance of the carrot seeder in terms of mean number of seeds planted in each hill, mean hill center of the seeds scattered or planted in each hill, Scattering Distance Ratio (SDR), mean hill spacing and percentage of missed hills were evaluated at 7 levels of operating speed. These parameters were analyzed through averaging and by considering the SD, CV, and analysis of variance. The hill center and missed hill percentage were significantly influenced by the operating forward speed (Table 3). However, the mean count of seeds dropped per hill, mean hill spacing, missed hills, and scattering distance ratio did not show a statistically significant relationship with respect to operating speed.

**Table 3:** The parameters used in the evaluation of the hill dropping uniformity of the seeder with corresponding p-values.

Parameters of hill dropping uniformity	*p* values
Mean number of seeds per hill	0.399310^ ns^
Mean hill center	0.019968**
Mean hill spacing	0.159232^ ns^
Scattering distance ratio	0.015242**
Missed hill, %	0.034314**

^ns^,**, nonsignificant or significant at the *p* ≤ 0.05, respectively.

### Number of seeds

The mean number of seeds deposited in each hill ranged from 2.8 to 4.0 (Table 4) which is within the desired number of seeds of 2 to 6 seeds per hill. Similarly, the mean number of discharged seeds was in accordance with the results from the evaluation of the same seeder administered by the Agricultural Machinery Testing and Evaluation Center under the Test Report No. 2020-0448^34^ and Test Report No. 2021-0025^31^. Analysis of variance showed that the differences in the mean number of seeds in each hill at all forward speeds were statistically insignificant although it was reduced at higher speeds. This indicates that the seed cell design was effective to load statistically uniform numbers of seeds which are likewise within the target number of seeds at all speeds. The number of seeds deposited in each hill measured through the coefficient of variation was highest (43.62%) at speed of 70 cm s^−1^ which gradually decreased to 21.7% at 48 cm s^−1^.

**Table 4:** The mean number of seeds deposited in each hill by the carrot seeder and the corresponding coefficient of variation as affected by operating speed.

Operating speed (cm s^−1^)	Mean seed number	CV, (%)
89	2.80	37.11
70	3.33	43.63
61	3.56	33.40
51	3.78	28.83
48	4.00	21.73
38	3.94	34.34
34	3.67	37.44

At 34 cm s^−1^, the CV was observed to increase to 37.44%. The relatively low speed could have caused the seeds in the hopper to behave such that the agitating force developed in the circular movement of the cylinder was not enough to allow the carrot seeds to flow into the seed cell. On the other hand, at slightly higher speeds where lower CVs were observed, the speed could have created enough vibration among the seeds that the cells were able to load seeds. At relatively high speeds like 70 and 89 cm s^−1^ with high CVs of 37.11 and 43.62%, the higher speed may have caused the seeds in the hopper to move in a “bombard” motion such that the loading time of the cell was not sufficient. Another factor is the hairy coating of carrot seeds that causes clustering, which may have also affected the seed loading capacity of the seed cell^4^.

### Hill center

**Figure 9:**
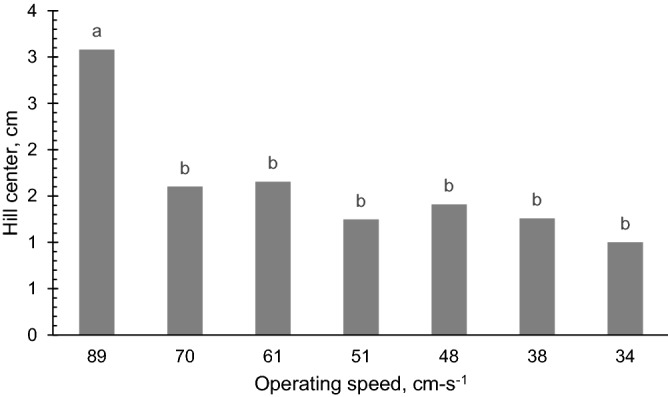
The influence of the different levels of operating speeds (cm s^−1^) on the hill center of carrot seeds. Operating speeds of different letters denote significant difference (*p* < 0.005) on the hill center.

The mean hill center varied with respect to operating speed (*p* < 0.05). Specifically, the hill center at speed 70 down to 34 cm s^−1^ were statistically the same with values ranging from 0.99 to 1.6 cm (Fig. 9).

At 89 cm s^−1^, hill center dramatically increased to 3.08 cm indicating that seeds are more dispersed at this speed compared to the other. The falling motion of a group of carrot seeds from the seed discharge point is governed by the law of the free-falling body where mass is the major consideration for a seed to either behave in a uniform path. In principle, if two seeds fall from the same origin at the same time, which is the seed cell in this case, given that they have the same mass, then it is expected that they will reach the ground at the same time and possibly at the same landing location. However, since carrot seeds are of tiny size and are varied in mass, the falling motion of each seed is not the same as the other. In addition, the seeds’ configuration in a seed cell follows a filling pattern wherein one seed has to fall first before the other. This is especially in the case of bigger seeds. The combined effect of these scenarios with the quick forward speed explains the higher values of hill center. Overall, as the forward speed decreases the hill center improves. This observation is supported by the findings of Karayel^9^. The aerodynamics governing seeds falling from a moving object may be used to further validate this observation.

The coefficient of variation of the mean hill center does not show a distinguishable trend with respect to speed (Table 5). The number of seeds deposited in each hill could have affected the high variability of the hill center at all speeds. One factor affecting this is the variable size of the carrot seeds where at some instances there were only a few seeds planted in a hill. When visually inspected, those hills with fewer seeds appeared to contain seeds of sizes higher than the average size. There were also hills that were sown with higher numbers of seeds. It was observed visually that the seeds in these hills appeared to be smaller than the average size of the carrot seed. Data relating to the size of the seed may still have to be validated in future studies. The same observation was reported by Gaikwad and Sirohi^35^. Furthermore, these smaller seeds were scattered at a wider range as indicated by higher hill centers compared to the larger ones, wherein they were clustered closer to each other.

**Table 5:** Mean values for the coefficient of variation of the hill center of the seeds in each hill as affected by the levels of operating speed.

Operating speed of the seeder (cm s^−1^)	CV (%)
89	58.44
70	62.45
61	63.34
51	68.55
48	59.44
38	76.23
34	57.57

### Hill spacing

**Figure 10:**
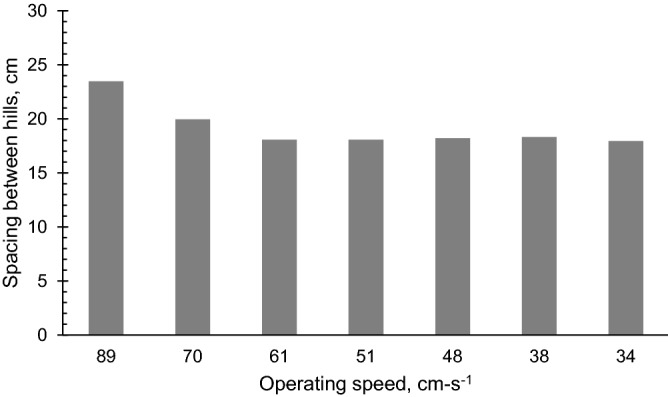
The influence of the different levels operating speeds (cm s^−1^) on the spacing between hills of deposited carrot seeds by the carrot seeder along the row.

The mean hill spacing, and the operating speed did not show any statistical relationship with respect to speed (Fig. 10). The coefficient of variation of the hill spacings as affected by the operating speed is presented in Table 6. The coefficient of variation increased considerably for an operating speed of 89 cm s^−1^.

**Table 6:** Mean values for the coefficient of variation of the spacing between hills of the seeds deposited by the carrot seeder as affected by the levels of operating speed.

Operating speed of the seeder, (cm s^−1^)	CV, (%)
89	25.83
70	14.13
61	14.19
51	9.60
48	7.79
38	11.43
34	7.39

A lower coefficient of variation of hill spacing was obtained at 70 cms^−1^ and below. The decrease in the uniformity of the hill spacing can be due to factors as described in previous studies. The high speed of the metering cylinder at the high operating speed could have resulted in an irregular distribution of seeds. Similarly, Karayel and Özmerzi^9^; Topakci et al.^32^; and Karayel^33^ observed that hill dropping uniformity of seeders differed most at faster seed metering speed. The same observation on higher variability of hill spacing at increased speed was reported by Badua et al.^36^ Pareek et al.^5^ concluded that the CV of the spacing of their seeder significantly increased at increased forward speed. Among the four levels of forward speed tested in their seeder, the highest CV (25.55%) was observed at the fastest speed of 33 m s^−1^. Virk et al.^37^, added that an increase in meter speed degrades the seed spacing uniformity as a result of their evaluation on two different types of seeders at 20 levels of meter speeds ranging at 15.4–43 RPM with CVs of 26.1–36.4%, respectively. Furthermore, the higher speed could have caused vibration of the seeds inside the cylinder causing the seeds to bounce and potentially affect the variability of seeds being dispensed into the ground. A similar result was reported by Mangus et al.^38^, namely that variability in plant spacing increased at faster planting speed.

### Scattering distance ratio

**Figure 11:**
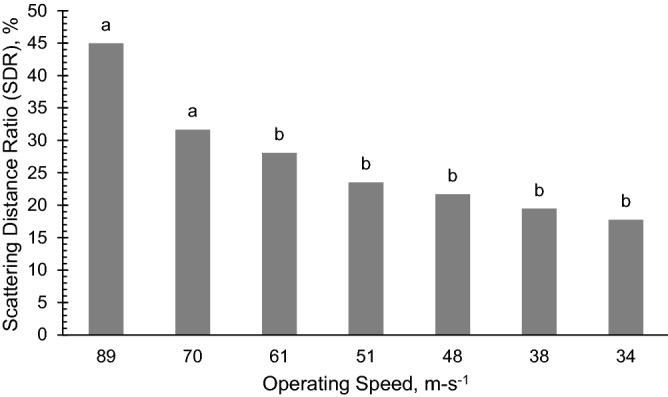
The influence of the different levels operating speeds (m s^−1^) on the Scattering Distance Ratio of deposited carrot seeds by the carrot seeder along the row. Operating speed with different letter denotes significant difference (*p* < 0.005) on the SDR.

The scattering distance ratio (SDR) of operating speeds of the seeder from 34 to 61 cm s^−1^ was 17.74–28.10% sufficient for hill dropping of carrot (Fig. 11).

This indicates that at 34–61 cm s^−1^, the seeds deposited in each hill were less scattered and that the seeder was able to deposit seeds at all hills at a uniform area coverage. SDRs in these speeds were lower than 30% which was far better than the roller seeder tested by Ryu and Kim^7^ at operating speeds of 20–90 cm s^−1^ with SDR values ranging from 76 to 115%. Nonetheless, since the scattering distance ratio of the carrot seeder was poorer at the higher operating speeds such as 31.6 and 44.97% were recorded at 70 and 89 cms^−1^ respectively, the effect of seed drop should be considered a critical factor. The higher operating speeds resulted in irregular falling velocities and trajectories of the seeds. Increasing operating speed increased the speed of seed moving in the direction of seeder travel, with an accompanying decrease in hill distance uniformity and increase in scattering distance ratio.

### Missed hills

The increase in operating speed significantly affected the ability of the seeder to deposit seeds at a regular interval (*p* < 0.05) (Table 7). Skips started to occur at 70 cm s^−1^ where the carrot seeder missed depositing seeds to 5.55% of the hills and increased to 16.67% at 89 cm s^−1^. The situation where seed cells failed to load with seeds at faster speeds could be that the cells did not experience sufficient time to load seeds during rotation. Singh et al.^19^; and Yazgi and Degirmencioglu^21^ also reported similar observations^19,21^. Kumar et al.^30^, claimed that fast rotation of a cylindrical drum causes seeds to fall in avalanches and that fast rotation could create centrifugal force that diverts the seeds out of the seed cell. The highest missed index of 22.0% occurred at the highest speed of sowing disc^39^. Kowalzuk et al.^40^ reported that among the different peripheral speeds of the sowing disc under laboratory test, 13.6% skips were observed at the highest speed (42 cm s^−1^) while 7.8% skips were recorded from the lowest speed (23 cm s^−1^)^39^.

**Table 7:** Mean values missed hills in percent as affected by the levels of operating speed.

Operating speed of the seeder (cm s^−1^)	Missed hills, %
89	16.67^a^
70	5.56^b^
61	0.00^c^
51	0.00^c^
48	0.00^c^
38	0.00^c^
34	0.00^c^

Means of the same letter do not differ significantly at 5% level of significance.

These missed hills explain why the mean spacing between hills at these speeds was higher than the hill spacings at operating speeds under 70 cm s^−1^ with a mean value of 19.94 cm and 23.46 cm, respectively, which is, on average, 22.62% higher than the designed hill spacing of 17.7 cm. Operating speeds under 70 cm s^−1^ were able to drop seeds at all hills.

## Conclusions

A carrot seeding machine was successfully designed, fabricated, and tested in this study. Results of evaluation of the influence of operating speed on the performance of the seeder revealed that the seeder is able to deposit 2–4 seeds at all speeds, which was within the design target of 2–6 seeds per hill. The hill center and coefficient of variation of hill spacing increased considerably for the highest operating speed of 89 cm s^−1^. Hill centers at operating speed of 70 cm s^−1^ and under, with values ranging from 0.9 to 1.6 cm, are statistically lower than at 89 cm s^−1^ which was 30.8 mm. This indicates that seeds planted at the highest speed are scattered to a wider area as compared to the seeds dropped at an operating speed of 70 cm s^−1^ where they are closer to each other and have a lower hill center. On the other hand, the spacing between hills wase not affected by the operating speed with values that ranged at 17.9–23.4 cm. The seeder maintained a uniform spacing between hills within this range. No missed hills were observed at operating speeds under 70 cm s^−1^. The scattering distance ratios for operating speeds from 34 to 61 cm s^−1^ were 17.74% to 28.09%; this range is adequate for hill dropping of carrot seeds.

## Supplementary Information

Supplementary Information.

## Data Availability

The data used in this study is available to the corresponding author upon request.

## List of symbols

$CV$: Coefficient of variation
$C_{i}$: Mean hill center
$$F_{f}$$: Frictional force
$i$: Transmission rate between wheel and metering cylinder
$g$: Acceleration due to gravity
$$l$$: Seed length
$L$: Length of cylinder
$$m_{s}$$: Seed mass
$m_{mc}$: Mass of metering component
$n_{i}$: Total number of seeds in a hill
$r$: Inner radius of metering cylinder
$R$: Outer radius of metering cylinder
$SDR$: Scattering distance ratio
$$SD$$: Standard deviation
$sh$: Slant height of seed cell
$S_{i}$: Distance between hills
$t$: Thickness of carrot seed
$t_{c}$: Thickness of cylinder
$V_{c}$: Volume of metering cylinder
$V_{s}$: Maximum volume of carrot seeds in the metering cylinder
$w$: Width of carrot seed
$X_{i, j}$: Position of the $i{\text{th}}$ carrot seed in the $j{\text{th}}$ hill
$\mu$: Static friction coefficient (SFC)
$\theta$: Angle of inclination of seed cell
$\overline{x}$: Theoretical spacing between hills
$\sigma \left( {SP} \right)$: Standard deviation of the distance between hills

## References

[1] Maghirang, R. G., Rodulfo, G. S. & Kebasen, B. *Carrot Production Guide*. 2 (2009).

[2] PAP, A. W. *Poland is the Third Largest Carrot Producer in the EU*. 2–5 (2021).

[3] DA FRO 2. Carrot Production Guide. *Dep. Agric.* **15**, 1–23 (2016).

[4] Philippine Statistics Authority (2020). Crops Statistics of the Philippines. Crops Stat. Philipp..

[5] Pareek CM, Tewari VK, Machavaram R, Nare B (2021). Optimizing the seed-cell filling performance of an inclined plate seed metering device using integrated ANN-PSO approach. Artif. Intell. Agric..

[6] Siemens MC, Gayler RR (2016). Improving seed spacing uniformity of precision vegetable planters. Appl. Eng. Agric..

[7] Ryu IH, Kim KU (1998). Design of roller type metering device for precision planting. Trans. Am. Soc. Agric. Eng..

[8] Sharipov GM, Paraforos DS, Pulatov AS, Griepentrog HW (2017). Dynamic performance of a no-till seeding assembly. Biosys. Eng..

[9] Karayel D (2009). Performance of a modified precision vacuum seeder for no-till sowing of maize and soybean. Soil Tillage Res..

[10] FAO. *Planting of KS Kuroda variety carrots to withstand heavy and prolonged rainfall, Philippines*. *Food and Agriculture Organization of the United Nations* https://www.fao.org/teca/es/technologies/7865 (2013).

[11] Yehia, I., Abd ElGawad, F. E., AL-Gezawe, A. & Altermezy, G. Development and Evaluation of A Carrot Seeder تطوير وتقييم آلة لزراعة بذور الجزر. *J. Soil Sci. Agric. Eng.* **12**, 509–518 (2021).

[12] Gautam A, Khurana R, Manes GS, Dixit AK, Verma A (2019). Development and evaluation of inclined plate metering mechanism for carrot (*Daucus Carota* L.) pelleted seeds. Int. J. Bio-resour. Stress Manag..

[13] Panning JW, Kocher MF, Smith JA, Kachman SD (2000). Laboratory and field testing of seed spacing uniformity for sugarbeet planters. Appl. Eng. Agric..

[14] Zhan Z, Yaoming L, Jin C, Lizhang X (2010). Numerical analysis and laboratory testing of seed spacing uniformity performance for vacuum-cylinder precision seeder. Biosys. Eng..

[15] Condra C, Buschermohle M, Hart W, Smith A (2017). Influence of planter width, planting speed, and perimeter-to-area ratio on Field efficiency for row crop planters. 2017 ASABE Annu. Int. Meet..

[16] Buitenwerf H, Hoogmoed WB, Lerink P, Müller J (2006). Assessment of the behaviour of potatoes in a cup-belt planter. Biosys. Eng..

[17] Ekka U, Singh N, Bharti N, Sahoo PK, Singh MK (2019). Design of seed metering system for jute seeds. Agric. Eng. Int. CIGR J..

[18] Bamgboye A, Mofolasayo A (2006). Performance evaluation of a two-row okra planter. CIGR Ejounal. Int. Agric. Environ. Eng..

[19] Singh RC, Singh G, Saraswat DC (2005). Optimisation of design and operational parameters of a pneumatic seed metering device for planting cottonseeds. Biosys. Eng..

[20] Özmerzi A, Karayel D, Topakci M (2002). Effect of sowing depth on precision seeder uniformity. Biosys. Eng..

[21] Yazgi A, Degirmencioglu A (2007). Optimisation of the seed spacing uniformity performance of a vacuum-type precision seeder using response surface methodology. Biosys. Eng..

[22] Karayel D, Wiesehoff M, Özmerzi A, Müller J (2006). Laboratory measurement of seed drill seed spacing and velocity of fall of seeds using high-speed camera system. Comput. Electron. Agric..

[23] Rasouli F, Sadighi H, Minaei S (2009). Factors affecting agricultural mechanization: A case study on sunflower seed farms in Iran. J. Agric. Sci. Technol..

[24] Kachman SD, Smith JA (1995). Alternative measures of accuracy in plant spacing for planters using single seed metering. Trans. ASAE.

[25] Aydin C (2003). Physical properties of almond nut and kernel. J. Food Eng..

[26] Shafaei SM, Nourmohamadi-Moghadami A, Kamgar S (2016). Analytical study of friction coefficients of pomegranate seed as essential parameters in design of post-harvest equipment. Inf. Process. Agric..

[27] Kaliniewicz, Z. *et al.* Effects of friction plate hardness and surface orientation on the frictional properties of cereal grain. *Int. J. Food Sci.* **2020**, (2020).

[28] Kaliniewicz Z, Markowski P, Andres A, Jadwisieńczak K (2015). Frictional properties of selected seeds. Tech. Sci..

[29] Xie F, Yu Q, Li X (2021). Design and analysis of self-propelled vegetable seeder. J. Phys. Conf. Ser..

[30] Prasanna Kumar GV, Srivastava B, Nagesh DS (2009). Modeling and optimization of parameters of flow rate of paddy rice grains through the horizontal rotating cylindrical drum of drum seeder. Comput. Electron. Agric..

[31] Agricultural Machinery Testing and Evaluation Center (AMTEC). *Test Report No. 2021-0025. Agricultural Machinery Seeder and Planter. SEEDTECH ST4–2*. www.amtec.ceat.uplb.edu.ph (2021).

[32] Karayel, D. Effect of forward speed on hill dropping uniformity of a precision vacuum seeder. **14**, 3–6 10.21273/HORTTECH.14.3.0364 (2004).

[33] Topakci M, Karayel D, Canakci M, Furat S, Uzun B (2011). Sesame hill dropping performance of a vacuum seeder for different tillage practices. Appl. Eng. Agric..

[34] Agricrultural Machinery Testing and Evaluation Center (AMTEC). *Test Report No. 2020–0448. Agricultural Machinery-Seeder and Planter. SEEDTECH ST4–1*. www.amtec.ceat.uplb.edu.ph (2020).

[35] Gaikwad BB, Sirohi NPS (2008). Design of a low-cost pneumatic seeder for nursery plug trays. Biosys. Eng..

[36] Badua SA, Sharda A, Strasser R, Ciampitti I (2021). Ground speed and planter downforce influence on corn seed spacing and depth. Precis. Agric..

[37] Virk SS, Fulton JP, Porter WM, Pate GL (2020). Row-crop planter performance to support variable-rate seeding of maize. Precis. Agric..

[38] Mangus DL, Sharda A, Zhang N (2016). Development and evaluation of thermal infrared imaging system for high spatial and temporal resolution crop water stress monitoring of corn within a greenhouse. Comput. Electron. Agric..

[39] Krzaczek P, Szyszlak J, Zarajczyk J (2006). Assessment of the influence of selected operating parameters of S071/B KRUK seeder on seeding Sida hermaphrodita Rusby seeds. Int. Agrophys..

[40] Kowalczuk, J.*et al.* Comparison of the Quality of Seeding the Hermaphrodita Rusby Seeds by S071 Kruk Seeder in Laboratory and Field Conditions. Paper presented at the VI International Scientific Symposium Farm Machinery and Processes Management in Sustainable Agriculture, Lublin, Poland. https://depot.ceon.pl/bitstream/handle/123456789/14642/FMPMSA2013_Proceedings.pdf?sequence=1&isAllowed=y (2013).

