# Publisher Correction: Investigation of the performance of a cylindrical hopper and metering device of a carrot seeder

**DOI:** 10.1038/s41598-023-29356-8

**Published:** 2023-02-10

**Authors:** Marvin T. Valentin, Andrzej Białowiec, Davut Karayel, Algirdas Jasinskas, Daniel Ciolkosz, Jeffrey A. Lavarias

**Affiliations:** 1 Wrocław University of Environmental and Life Sciences, Department of Applied Bioeconomy, 25th Norwida Str., 51‑630 Wrocław, Poland; 2 Associate Member, Engineering and Industrial Research, National Research Council of the Philippines, Department of Science and Technology, Taguig, Philippines; 3 Benguet State University, Km. 5, La Trinidad, 2601 Benguet, Philippines; 4 Department of Agricultural Engineering and Safety, Vytautas Magnus University, Agriculture Academy Studentu 15A, 53362 Akademija, Kaunas Reg. Lithuania; 5 Faculty of Agriculture, Department of Agricultural Machinery and Technologies Engineering, Akdeniz University, 07070 Antalya, Turkey; 6 Department of Agricultural and Biological Engineering, Pennsylvania State University, University Park Campus, State College, USA; 7 Department of Agricultural and Biosystems Engineering, College of Engineering, Central Luzon State University, Science City of Muñoz, Nueva Ecija, Philippines

Correction to: *Scientific Reports* https://doi.org/10.1038/s41598-022-25798-8, published online 16 January 2023

In the original version of this Article Andrzej Białowiec was incorrectly affiliated with ‘Department of Agricultural Engineering and Safety, Vytautas Magnus University, Agriculture Academy Studentu 15A, 53362, Akademija, Kaunas Reg., Lithuania’. The correct affiliation is listed below.

Wrocław University of Environmental and Life Sciences, Department of Applied Bioeconomy, 25th Norwida Str., 51-630, Wrocław, Poland

The original Article and accompanying Supplementary Information file have been corrected.

