# Supplementary material for: Investigation of the performance of a cylindrical hopper and metering device of a carrot seeder
Source: Sci Rep. 2023 Jan 16;13:813. doi: 10.1038/s41598-022-25798-8 (PMC9842741; doi:10.1038/s41598-022-25798-8)
Supplement: Supplementary file 1 — Supplementary Information. [file 41598_2022_25798_MOESM1_ESM.pdf]

Investigation of the Performance of a Cylindrical Hopper and Metering Device of a Carrot Seeder

Marvin T. Valentin[1,2,3], Andrzej Białowiec[1], Davut Karayel[4,5], Algirdas Jasinskas[4], Daniel Ciolkosz[6], Jeffrey A. Lavarias[7]

[1] Wrocław University of Environmental and Life Sciences, Department of Applied Bioeconomy, 25th Norwida Str. 51-630 Wrocław, Poland; marvin.valentin@upwr.edu.pl
[2] Associate Member, Engineering and Industrial Research, National Research Council of the Philippines, Department of Science and Technology, Taguig, Philippines
[3] Benguet State University, Km. 5, La Trinidad, 2601 Benguet, Philippines; m.valentin@bsu.edu.ph
[4] Department of Agricultural Engineering and Safety, Vytautas Magnus University, Agriculture Academy Studentu 15A, LT-53362, Akademija, Kaunas Reg., Lithuania; algirdas.jasinskas@vdu.lt
[5] Faculty of Agriculture, Department of Agricultural Machinery and Technologies Engineering, Akdeniz University, 07070, Antalya, Turkey; dkarayel@akdeniz.edu.tr
[6] Department of Agricultural and Biological Engineering, Pennsylvania State University, University Park Campus, State College, USA; dec109@psu.edu
[7] Department of Agricultural and Biosystems Engineering, College of Engineering, Central Luzon State University, Science City of Muñoz, Nueva Ecija, Philippines; jeffreylavarias@clsu.edu.ph
* Corresponding author:

Supplementary

1. Existing plant bed setup

The current practice in planting carrots in the place of study is to create plant beds of definite size. In between the plant beds are spaces called by “kankana-ey” farmers in the Province of Benguet as “kanal” that can be translated in English as canal. The canal serves as passage for crop establishment operations such as side dressing, thinning, fertilizer application and most importantly better aeration purposes. The width (S_{PB}) of the canal is usually 10 to 20 cm. In this study, 10 cm was used which is also enough to accommodate the ground wheel of the carrot seeder. Shown in Figure 1 is a sample layout of a plant bed for carrot production. Considering the distribution of the carrot seeds over the plant bed, there are some measures that have to be observed. These are the row spacing (RS) and hill spacing (HS) and the space between the edge of the plant bed to the carrot seeds.

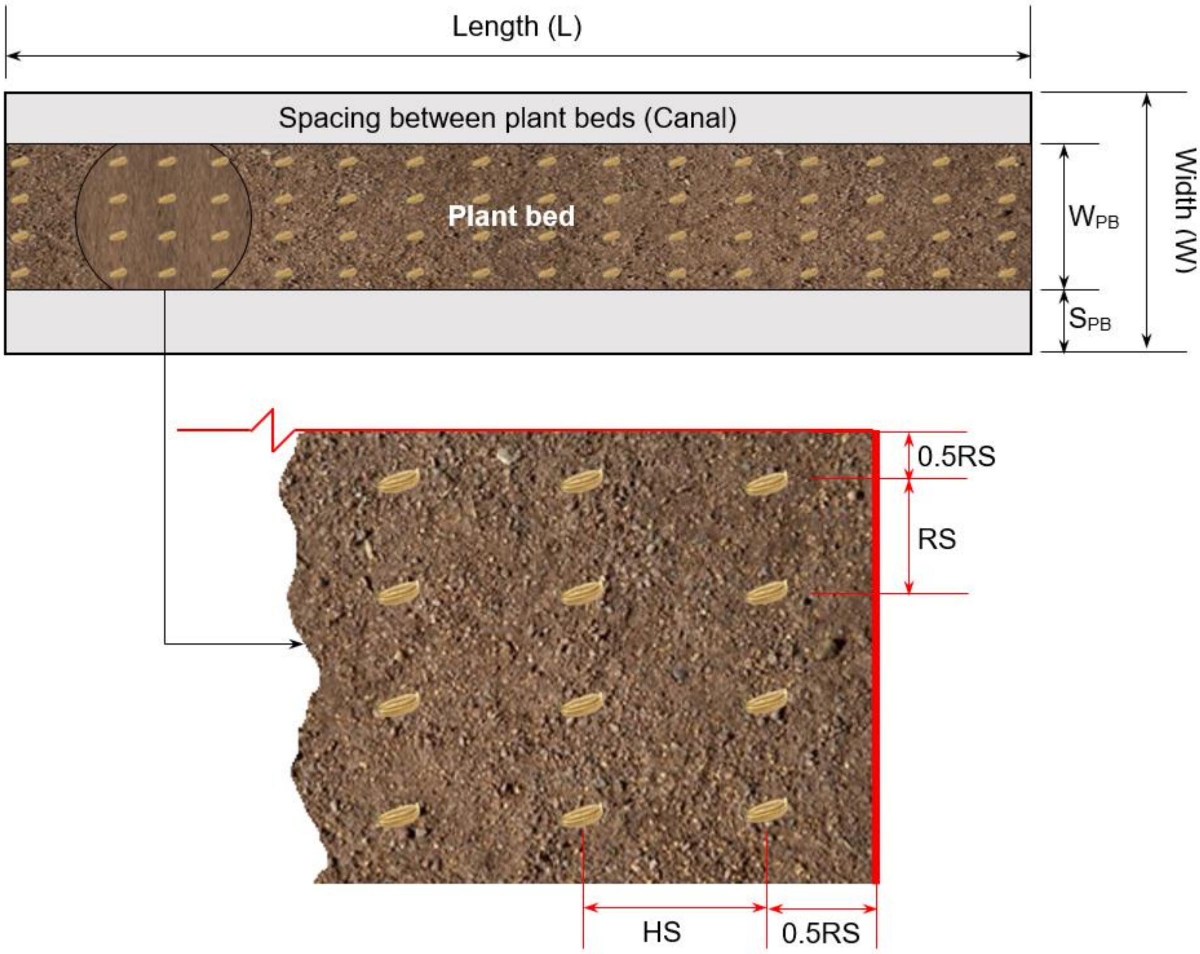

Figure S 1. Typical plant bed setup for carrot production.

2. Calculation

The calculation and basis of the number of seeds in each hill is presented herein. In Figure 1 consider a farm with a size of 1.0 hac comprised of several plant beds of the same size. For calculation purposes, assume that those plant beds are joined together to form a very long plant bed with total area including the area of the canal to be 1.0 hac, then the total area for the plant bed which is the Total Effective Area (TEA) and the total area for the canal can be distinguished.

2.1. **Total Effective Area (TEA).** The total area is 1.0 hac while the design plant bed width (W_{PB}) is 20.0 cm and the width of the canal or space between plant bed (S_{PB}) is 10.0 cm. Thus, TEA is

$$Total\ Area = 10{,}000.00\ m^2$$
$$10{,}000.00\ m^2 = L\ x\ (W_{PB} + 2S_{PB})$$
$$10{,}000.00\ m^2 = L\ x\ (0.20\ m + 2x0.10\ m)$$
$$L = 25{,}000\ m$$
$$TEA = L\ x\ W$$

$$TEA = 25{,}000.00\, m\, x\, 0.20\, m$$
$$= 5{,}000.00\, m^2$$

2.2. **Total number of hills in a hectare (H_T).** The total number of hills in relation to the total effective area can be calculated as follows. In the 1 hectare, only the TEA is planted with seeds since some area is dedicated for canal as described earlier. The area per hill A_H can be determined by the product of the row and hill spacing used in the study which are 5.0 cm and 17.7 cm, respectively.

$$H_T = \frac{Total\ effective\ area}{A_H}$$
$$= \frac{5{,}000.00\, m^2}{0.05\, m\, x\, 0.177 m}$$
$$= 564{,}971.75\ \ hills\ per\ hac$$

2.3. **Seeds per hectare**. The density of seeds used in this study is 2.2 g per 1000 seeds. The recommended plant density is 6 kg-hac^{-1}. Thus, the total seeds needed in a hectare (SPH) is:

$$SPH = \frac{6000 g/hac}{2.2 g/1000 seeds}$$
$$= \ 2{,}727{,}272.73\ \ seeds/hac$$

2.4. **Seeds per hill**. Therefore, the seeds needed in each hill can be further calculated as:

$$Seeds_{Hill} = \frac{2{,}727{,}272.73\ \ seeds/hac}{564{,}971.75\ hills\ per\ hac}$$
$$= 4.83\ seeds$$

Considering the germination rate of 80% (usually included in the seed information upon purchasing), the total number of seeds to be planted in each hill should be:

$$Seeds_{Hill} = \frac{4.83\ seeds}{0.80}$$
$$= 6\ seeds/hill$$

3. Working equation.

For future references, the working equation to determine the number of seeds per hill, considering the plant density per area, hill and row spacing and germination rate are known the following equations are proposed. Eq. 1 is the generic equation for the calculation of the seeds to be planted in a hill.

$$Seeds_{Hill} = \frac{SPH}{GR\ x\ H_T} \quad (1)$$

Where, SPH is the seed per hectare, GR is the germination rate, and H_T is the total hills in a hectare. The total number of seeds that can be planted in a hectare can be estimated in relation to the seeds bulk density and the recommended plant density (Eq. 2).

$$SPH = \frac{PD}{\rho_{BS}} \quad (2)$$

Where, PD is the plant density, kg-hac^{-1} and ρ_{BS} is the bulk density of the carrot seed. In this study it was 2.2g/1000seeds determined in the laboratory. The total number of hills can also be expounded by considering the ratio of the total effective area and the area per hill (Eq. 3).

$$H_T = \frac{TEA}{APH} \quad (3)$$

Where, H_T is total number of hills in a hectare, TEA is the total effective area in a hectare, and APH is the area per hill. Substituting Eq. 3 and Eq. 2 into 1 and simplifying gives Eq. 4 which is now the working equation relating the plant density, area of each hill, germination rate, bulk density of the carrot seeds and total effective area to estimate the number of seeds in a hill.

$$Seeds_{Hill} = \frac{PD\ x\ APH}{GR\ x \rho_{BS}\ x\ TEA} \quad (4)$$

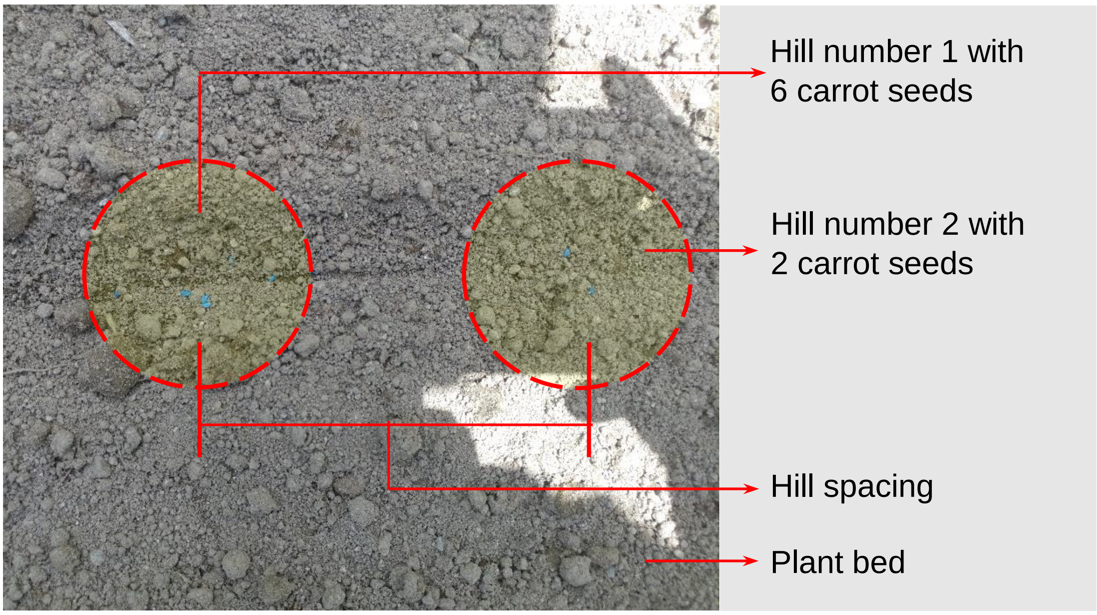

Figure S 2. Picture of actual carrot seeds planted by the carrot seeder on a plant bed during on the of the field evaluations. Those blue particles are the carrot seeds enclosed in the red circle.

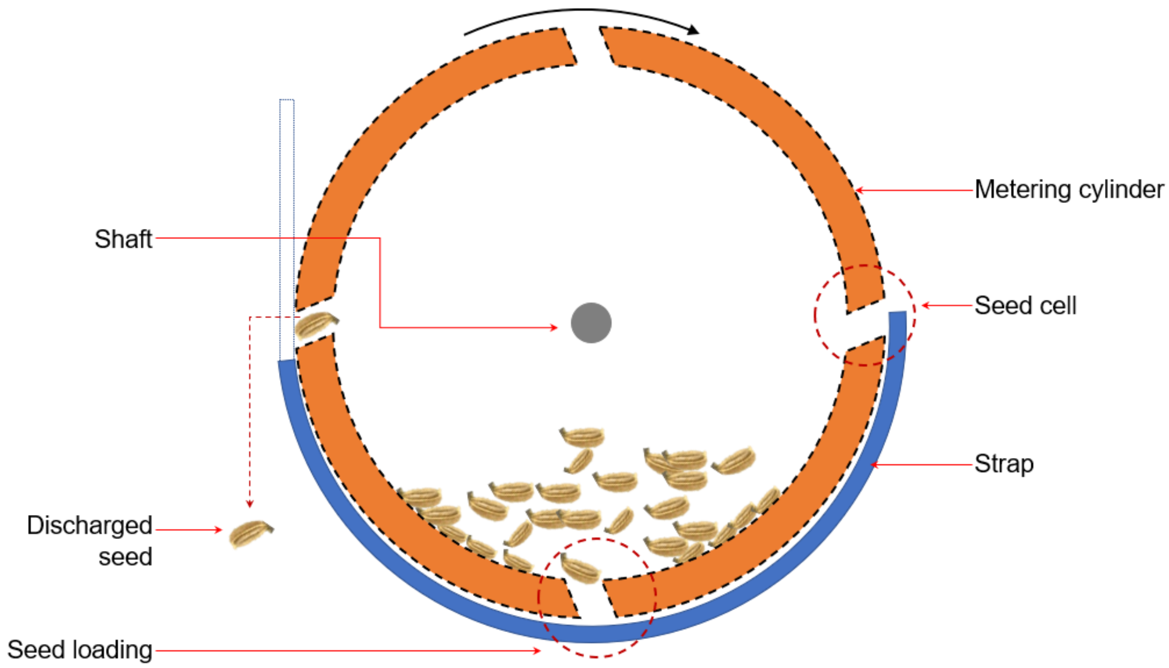

Figure S 3. Seeding mechanism in which the metering cylinder, attached to a shafting, rotates to allow the seed loading by means of gravity into the seed cell then discharges at the discharge point after the seed cell surpasses the strap.
